# Machine learning–derived intracellular protein–based prognostic model reflecting a senescence-associated secretory phenotype-driven immunosuppressive microenvironment in lung adenocarcinoma

**DOI:** 10.7717/peerj.21835

**Published:** 2026-10-21

**Authors:** Xiaoning Chang, Lihong Jiang, Lingxiao Zhang, Tong Ren, Chaoxin Fan, Pei Li

**Affiliations:** 1 Department of Oncology, Xi’an Chang’an District Hospital, Xi’an, Shaanxi, China; 2 Department of Pathology, Xi’an Chang’an District Hospital, Xi’an, Shaanxi, China; 3 Department of Medical Oncology, The First Affiliated Hospital of Xi’an Jiaotong University, Xi’an, Shaanxi, China; 4 Department of Nuclear Medicine, Xi’an No. 3 Hospital, The Affiliated Hospital of Northwest University, Xi’an, Shaanxi, China; 5 Department of Oncology, Xi’an People’s Hospital (Xi’an Fourth Hospital), Xi’an, Shaanxi, China; 6 Department of Respiratory Medical Oncology, Shandong First Medical University and Shandong Academy of Medical Sciences, Shandong Cancer Hospital and Institute, Jinan, Shandong, China

**Keywords:** Intracellular protein networks, Senescence-associated secretory phenotype, Tumor immune microenvironment, Machine learning, Predictive model

## Abstract

**Background:**

This study aimed to investigate the associations of senescence-associated secretory phenotype (SASP)-related genes with the tumor immune microenvironment (TIME) and clinical outcomes in lung adenocarcinoma (LUAD) and to develop and evaluate a prognostic signature for risk stratification and exploratory treatment-response prediction.

**Methods:**

Non-negative matrix factorization (NMF) was conducted to identify distinct molecular subtypes. Least absolute shrinkage and selection operator (LASSO), Random Forest, and extreme gradient boosting (XGBoost) were utilized to identify core prognostic SASP genes. The expression patterns of selected SASP-related genes were explored using multi-omics datasets, LUAD cell lines, and clinical specimens.

**Results:**

Cluster 1 was associated with significantly shorter overall survival (OS), enhanced senescence pathway activity, and computationally inferred immunosuppressive and immune-exclusion features. Five intracellular SASP regulators—*ANGPTL4*, *CDKN2A*, *H2AX*, *MMP1*, and *CCL20*—were selected through machine learning to construct the SASP Protein Regulator Score (SASP-PRS). A high SASP-PRS was associated with the activation of senescence/SASP and cell-cycle pathways, suppression of antitumor immune signatures, and distinct immune-checkpoint and chemokine expression profiles. A higher SASP-PRS was also associated with advanced disease stage, poorer OS, predicted lower responsiveness to immunotherapy, lower predicted sensitivity to erlotinib, and higher predicted sensitivity to selected chemotherapeutic agents. The nomogram incorporating the SASP-PRS demonstrated discriminatory ability and calibration for predicting 1-, 3-, and 5-year OS in the analyzed LUAD cohorts.

**Conclusion:**

These findings suggest that SASP-related gene-expression patterns are associated with LUAD molecular subtypes, senescence signaling, computationally inferred immune features, and treatment-related outcomes. The SASP-PRS may serve as a potential biomarker for prognostic stratification in LUAD. However, its utility for treatment-response assessment remains exploratory. Prospective external validation and functional studies are required to establish its clinical applicability and clarify the mechanisms linking SASP-related networks to the LUAD immune microenvironment.

## Introduction

Lung adenocarcinoma (LUAD), the most common subtype of non-small cell lung cancer (NSCLC), accounts for approximately 40% of all lung cancer diagnoses and remains a leading cause of cancer-related mortality worldwide, with over 1.8 million deaths annually (Siegel et al., 2025). Therapeutic advances—including targeted therapies against oncogenic drivers and immune checkpoint inhibitors (ICIs) targeting programmed death-1 (PD-1), programmed death-ligand 1 (PD-L1), and cytotoxic T-lymphocyte–associated protein 4 (CTLA-4)—have markedly improved outcomes for selected patients (Hirsch et al., 2022; Hendriks et al., 2024). Among these, ICIs have revolutionized the treatment landscape by enhancing the immune system’s ability to target and destroy tumor cells. However, response rates to ICIs remain limited, with only 15%–40% of patients achieving durable outcomes (Hegde & Chen, 2020). Resistance to immunotherapy, both intrinsic and acquired, poses a significant challenge. Increasing evidence suggests that the tumor immune microenvironment (TIME) plays a critical role in mediating resistance and modulating therapy outcomes (Liu et al., 2024). This underscores an urgent need to better understand the molecular and cellular factors influencing the TIME to optimize immunotherapy strategies in LUAD.

Cellular senescence, defined by irreversible cell cycle arrest due to various stresses such as oncogenic signaling and DNA damage, is a key feature of cancer biology (Liu et al., 2025). Senescent cells secrete a complex array of factors referred to as the senescence-associated secretory phenotype (SASP), which includes cytokines, chemokines, growth factors, and matrix metalloproteinases (Wang et al., 2024; Dong et al., 2024). SASP plays a dual role in regulating the TIME, with both antitumorigenic and protumorigenic effects. On one hand, SASP recruits immune cells such as T cells, natural killer (NK) cells, and dendritic cells (DCs), facilitating the clearance of senescent tumor cells (Kubuschok et al., 2006). On the other hand, SASP can drive chronic inflammation, promote immune escape, and reshape the microenvironment to favor tumor progression (Zhang et al., 2024). For example, SASP factors such as IL-6 and IL-8 have been implicated in recruiting immunosuppressive myeloid-derived suppressor cells (MDSCs) and regulatory T cells (Tregs), while also supporting tumor growth and metastasis (Ortiz-Montero, Londoño Vallejo & Vernot, 2017; Ruhland et al., 2016). Moreover, SASP can antagonize the efficacy of ICIs by promoting an immunosuppressive TIME, downregulating T cell activation, and altering checkpoint molecule expression (Du et al., 2024). These observations suggest that targeting SASP-related pathways could enhance immunotherapy efficacy. While recent studies have highlighted individual SASP-related genes as potential biomarkers (Lee et al., 2024), a comprehensive characterization of SASP gene signatures and their prognostic value remain unexplored in LUAD.

Advances in computational biology and multi-omics analysis have enabled the integration of bulk transcriptomic, proteomic, and single-cell RNA-sequencing (scRNA-*seq*) data to investigate molecular regulators of complex biological processes. Machine learning techniques have further refined biomarker discovery and risk prediction models (Sharma et al., 2024). Applying these technologies to analyze the SASP offers a promising approach to dissect its diverse roles in LUAD and its implications for prognosis and therapy. In this study, we systematically investigated SASP-related genes in LUAD using bioinformatics and machine learning approaches. Our objectives were to identify prognostically relevant SASP genes, characterize their associations with the TIME, and develop and evaluate a SASP Protein Regulator Score (SASP-PRS) for prognostic risk stratification and exploratory treatment-response assessment. We further characterized selected SASP-related genes using transcriptomic, proteomic, scRNA-*seq*, cell-line, and clinical-specimen data. The prognostic performance of the SASP-PRS was evaluated in LUAD-specific cohorts, whereas its associations with immunotherapy-related outcomes were explored using supportive external cohorts comprising patients with advanced NSCLC or urothelial carcinoma. These analyses aimed to provide a basis for subsequent prospective validation and mechanistic investigation of SASP-related networks in LUAD.

## Materials & Methods

### Publicly available data collection and preprocessing

Bulk RNA-sequencing (RNA-*seq*) analyses were conducted using the The Cancer Genome Atlas Lung Adenocarcinoma (TCGA-LUAD) cohort (https://portal.gdc.cancer.gov) and the GSE13213 dataset (https://www.ncbi.nlm.nih.gov/geo/). GSE13213, comprising 117 patients with LUAD, was used as an independent LUAD-specific bulk-transcriptomic validation cohort. For the TCGA-LUAD cohort, RNA-*seq* data in FPKM format and the corresponding clinical information were downloaded from the Genomic Data Commons (GDC) portal. The preprocessing steps for GSE13213 involved gene annotation using the GPL6480 platform to ensure accurate mapping within the expression matrix.

To explore the association between the SASP-PRS and immunotherapy-related outcomes, we included the IMvigor210 cohort (Rosenberg et al., 2024), which contains normalized gene expression and clinical response data from patients with metastatic urothelial carcinoma treated with the anti-PD-L1 antibody atezolizumab. The processed IMvigor210 data were obtained using the IMvigor210CoreBiologies R package and were originally generated in the IMvigor210 phase II trial (EGA: EGAS00001002556; https://ega-archive.org/. Because this cohort comprised patients with urothelial carcinoma rather than LUAD, it was used as a supportive pan-tumor immunotherapy cohort and not as LUAD-specific validation. In addition, GSE135222 was included as a supportive external NSCLC immunotherapy cohort. This dataset contains 27 advanced NSCLC patients treated with anti-PD-1/PD-L1 therapies and was used to assess the association of SASP-PRS with immunotherapy-related outcomes in NSCLC (Kim, Choi & Jung, 2020). Because the cohort was small and was not restricted to LUAD by histological subtype, it was not considered an independent LUAD-specific immunotherapy validation cohort. Proteomics data with clinical annotations were obtained from the Clinical Proteomic Tumor Analysis Consortium (CPTAC, https://proteomic.datacommons.cancer.gov/pdc) portal for protein-level validation of SASP regulators and the SASP-PRS.

Single-cell RNA sequencing (scRNA-seq) datasets (GSE117570 (https://www.ncbi.nlm.nih.gov/geo/), EMTAB6149 (https://www.ebi.ac.uk/biostudies/arrayexpress/studies/E-MTAB-6149), and GSE127465 (https://www.ncbi.nlm.nih.gov/geo/)) (Wang et al., 2021; Zilionis et al., 2019) were acquired to characterize SASP heterogeneity and immune context at cellular resolution. These datasets were derived from NSCLC cohorts not selected by histological subtype; they are used for supportive cellular-level characterization rather than LUAD-specific validation. A summary of all datasets used in this study, including their purposes, sample sizes, and key characteristics, is provided in Table S1. SASP-related genes were curated from the Molecular Signatures Database (MSigDB) and a previously published study. Specifically, 112 genes were obtained from the Reactome gene set REACTOME_SENESCENCE_ASSOCIATED_SECRETORY_PHENOTYPE_SASP in MSigDB, and an additional 83 SASP-related genes were collected from the literature (Saul et al., 2022). After merging the two gene lists and removing duplicates, a total of 191 unique SASP-related genes were retained for subsequent differential expression and downstream analyses.

### Identification of differential SASP genes and functional enrichment analysis

To identify SASP genes with significant differential expression between LUAD and normal lung tissues, differential expression analysis was conducted using the “limma” R package. SASP genes were considered significantly differentially expressed if they met the criteria of an adjusted *P*-value < 0.05 and an absolute log2 fold change ≥ 1. Functional enrichment analysis of the identified differentially expressed SASP genes was performed using the “clusterProfiler” R package. Gene Ontology (GO) analysis and Kyoto Encyclopedia of Genes and Genomes (KEGG) analyses were undertaken to identify key signaling pathways associated with the differentially expressed SASP genes.

### Determination and analysis of LUAD molecular subtypes based on SASP genes

Non-negative Matrix Factorization (NMF) was employed to stratify LUAD patients into distinct molecular subtypes based on SASP genes through the “NMF” R package. The optimal number of clusters (rank) was determined by iteratively minimizing the cophenetic correlation coefficient. Patients were assigned to specific subtypes using the resulting basis and coefficient matrices from the NMF algorithm. The prognostic significance of the molecular subtypes was assessed through survival analysis. To explore the biological diversity among the SASP-based LUAD molecular subtypes, Gene Set Enrichment Analysis (GSEA) was conducted using the “clusterProfiler” R package. Pathways meeting the threshold of adjusted *P*-values < 0.25 were considered significantly enriched. Additionally, single-sample GSEA (ssGSEA) was performed to investigate immune microenvironment differences across the LUAD subtypes by calculating enrichment scores for immune-related gene sets.

### Identification of core SASP genes and calculation of SASP protein regulator score

To identify core SASP genes, a comprehensive machine learning framework was employed, integrating Least Absolute Shrinkage and Selection Operator (LASSO) regression, Random Forest, and XGBoost, using the “glmnet”, “randomForest”, and “xgboost” R packages, respectively. LASSO regression identified features by shrinking coefficients to zero based on a penalty parameter optimized *via* tenfold cross-validation. In parallel, Random Forest ranked genes based on measures such as mean decrease in accuracy or Gini index, while XGBoost evaluated feature importance through performance-driven scores. The outputs of all three methods were intersected to compile a consensus list of core SASP genes, ensuring robustness and minimizing algorithm-specific biases.

SASP-PRS for each sample was calculated using a multivariate Cox regression model *via* the “survival” package in R software. The expression levels of the core SASP genes were weighted by their respective Cox regression coefficients, generating a cumulative score as follows:

\begin{eqnarray*}SASP-PRS=\sum _{i=1}^{n}(Expression~Level~of~{Gene}_{i} \times Coef{ }_{i}). \end{eqnarray*}

Z-score normalization was applied to standardize these scores. Samples with z-scores above zero were classified into the high-SASP group, and those below or equal to zero into the low-SASP group. Kaplan–Meier survival analysis and log-rank tests validated the stratification’s prognostic relevance. Time-dependent receiver operator characteristics (ROC) curve analysis was conducted to assess the model’s predictive ability and its utility for patient prognostic evaluation.

### Establishment and evaluation of predictive models

Predictive models were developed to estimate overall survival (OS) at different time points in LUAD patients by integrating clinical variables and SASP-PRS. Significant predictors of OS were identified through univariate and multivariate Cox regression analyses. Variables achieving statistical significance in the univariate analysis were incorporated into the multivariate analysis to refine the model and ensure its robustness. A prognostic nomogram was constructed using the “rms” R package to visually represent the predictive model, allowing for individualized OS predictions. Concordance Index (C-index) and the Area Under the Curve (AUC) value were calculated to measure its discriminative ability. Calibration curves were generated to evaluate the agreement between predicted and observed survival probabilities. Decision Curve Analysis (DCA) was conducted to further assess the clinical utility of the predictive models.

### Characterization of the immune microenvironment, biological processes, and response to anti-tumor therapy

The immune microenvironment was characterized by estimating immune cell infiltration levels using single-sample Gene Set Enrichment Analysis (ssGSEA), the MCPcounter algorithm (Becht et al., 2016), and the ImmuCellAI algorithm (Miao et al., 2020). For each immune cell type, normalized enrichment scores were calculated across individual samples or molecular clusters. Correlation analysis was conducted to evaluate the association between SASP-PRS and the expression levels of key immunological markers, including chemokines and immune checkpoint genes. GSEA was performed to further explore the biological processes correlated to the SASP-PRS. Pathways with adjusted *P*-values < 0.25 were considered statistically significant, providing functional insights into the molecular features of the SASP phenotype. To evaluate the therapeutic implications of the SASP phenotype, we predicted drug responses using the “pRRophetic” R package (Geeleher, Cox & Huang, 2014). Predicted half-maximal inhibitory concentration (IC50) values were calculated for each sample.

### Single-cell transcriptomic analysis

For scRNA-*seq* analysis, GSE117570 (Wang et al., 2021), EMTAB6149 (Lambrechts et al., 2018), and GSE127465 (Zilionis et al., 2019) datasets were processed in Seurat with standard QC and normalization. Cells with fewer than 200 detected genes, genes expressed in fewer than three cells, or mitochondrial gene proportion >10% were removed. Data were log-normalized and scaled to account for library size and mitochondrial content. Principal Component Analysis (PCA) was used for dimensionality reduction; clusters were identified *via* a Shared Nearest Neighbor (SNN) graph and visualized with t-SNE. Epithelial cells were classified as benign or malignant using CopyKAT based on inferred copy number variation profiles (Gao et al., 2021). SASP activity at single-cell resolution was quantified using Seurat’s AddModuleScore with curated SASP gene sets.

### RT-PCR

The human bronchial epithelial cell line BEAS-2B (ATCC), LUAD cell lines H1975 and A549 (ATCC), and the NSCLC cell line H1299 (ATCC) were cultured in DMEM high glucose (Cat#11965092; Gibco) or RPMI-1640 (Cat#11875093; Gibco) with 10% FBS (Cat#16000044; Gibco) and 1% Penicillin–Streptomycin (100 ×; Cat#15140122; Gibco) at 37 °C in a humidified incubator with 5% CO_2_. Total RNA was isolated with TRIzol Reagent (Cat#15596026; Invitrogen) per the manufacturer’s instructions. cDNA was synthesized from 1 µg RNA using the PrimeScript RT Reagent Kit with gDNA Eraser (Takara Bio, Cat#RR047A). Quantitative polymerase chain reaction (qPCR) was performed on a CFX96 system (Bio-Rad) using ChamQ Universal SYBR qPCR Master Mix (Vazyme, Cat#Q711). Primer sequences for *ANGPTL4*, *CCL20*, *H2AX*, *MMP1*, and *CDKN2A* were designed as shown in Table S2. Gene expression levels were normalized to GAPDH and calculated using the 2^ΔΔCT^ method. All RT-qPCR experiments were independently repeated three times using biological replicates.

### Immunohistochemistry

Tumor and adjacent normal lung tissues were collected from LUAD patients undergoing surgical resection at Shandong Cancer Hospital with approval from the Ethics Committee of Shandong Cancer Hospital and Institute (Ethical approval No. SDTHEC202603333). Individual consent for this retrospective analysis was waived. Specimens were fixed in 10% neutral-buffered formalin, embedded in paraffin, and sectioned at 4 µm thickness for immunohistochemical analysis. Tissue sections were incubated overnight at 4 °C with primary antibodies targeting ANGPTL4 (Cat# DF6751, 1:200; Affintiy), CCL20 (Cat# DF2238, 1:100; Affintiy), H2AX (Cat# GB111841, 1:200; Servicebio), MMP1 (Cat# GB115708, 1:1000; Servicebio), and CDKN2A (Cat# GB111143, 1:1000; Servicebio). Images were acquired using ZEISS Automatic Digital Slide Scanner. Immunohistochemical staining was semi-quantitatively scored according to staining intensity and the proportion of positive cells. Staining intensity was graded as 0 (negative), 1 (weak), 2 (moderate), or 3 (strong). The proportion of positive cells was scored as 0 (<5%), 1 (5%–25%), 2 (26%–50%), 3 (51%–75%), or 4 (>75%). The final Immunohistochemistry (IHC) score was calculated by multiplying the staining intensity score by the positive-cell proportion score, resulting in a total score ranging from 0 to 12. A total of six LUAD tumor tissue samples and six adjacent normal lung tissue samples were included for immunohistochemical staining.

### Statistical analysis

All statistical analyses were performed using R software (version 4.4.1; R Core Team, 2024). Correlations between continuous variables, such as SASP scores and immune gene expression levels, were analyzed using Pearson or Spearman correlation coefficients, depending on the data distribution. Assumptions of normality were assessed prior to selecting the appropriate correlation method. Survival analyses were conducted using the “survival” R package. Kaplan–Meier survival curves were generated to evaluate differences in OS between patient groups or molecular subtypes, and statistical significance was determined using log-rank tests. To account for potential confounding factors, multivariate Cox proportional hazards regression was performed. Adjusted HRs with corresponding 95% CIs were calculated to quantify the strength and direction of associations between variables and OS. This comprehensive approach ensured robust statistical validation of the study findings.

## Results

### Distinct immunological and biological characteristics of SASP subtypes in LUAD

The workflow of this study is illustrated in Fig. 1A. A total of 36 SASP-related genes were identified as differentially expressed between LUAD and normal lung tissues (Figs. 1B, 1C). Functional enrichment analysis revealed that these genes were primarily associated with processes such as cell chemotaxis, leukocyte chemotaxis, chromatin assembly and organization, neutrophil extracellular trap formation, cytokine-cytokine receptor interaction, and the IL-17 and TNF signaling pathways (Figs. 1D, 1E). Using the NMF algorithm, we identified two distinct molecular subtypes (Fig. 1F and Fig. S1). Survival analysis demonstrated that patients in Cluster 1 exhibited significantly poorer OS compared to those in Cluster 2 (Fig. 1G). Additionally, a higher proportion of advanced-stage disease was observed in Cluster 1 (Fig. 1H). GSEA showed Cluster 1 enriched for senescence-related pathways and cell-cycle processes (Fig. 2A), whereas gene sets related to T-cell activation and antitumor immune responses showed lower enrichment in Cluster 1 than in Cluster 2 (Fig. 2B). These transcriptomic features were consistent with a potentially less active antitumor immune state in Cluster 1. ssGSEA revealed lower estimated enrichment of CD8^+^ T cells, TILs, and DCs, and a reduced type II IFN response score in Cluster 1 (Figs. 2C, 2D). Consistently, MCPcounter provided computational estimates of lower T-cell abundance in Cluster 1 (Figs. 2E, 2F). Collectively, these findings indicate that SASP-related gene-expression patterns distinguish two LUAD subtypes with different survival, biological-pathway, and computationally inferred immune characteristics. Cluster 1 was associated with enhanced senescence and cell-cycle signatures and with immune features suggestive of a relatively immunosuppressive microenvironment.

**Figure 1:**
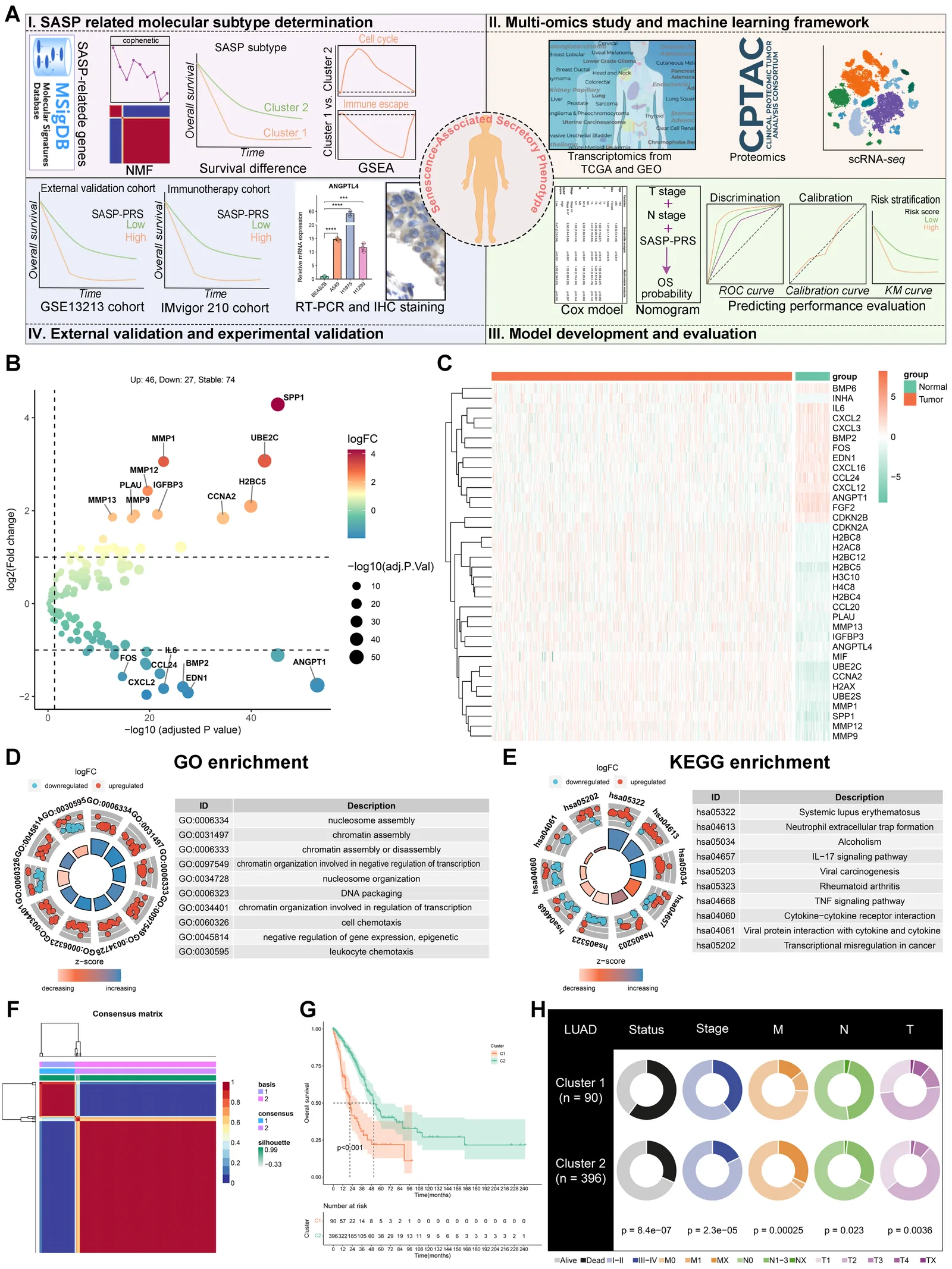
Analysis of senescence-associated secretory phenotype (SASP) genes in lung adenocarcinoma (LUAD). (A) Overview of the study design and methodology. (B–C) Differential expression analysis visualized by a volcano plot (B) and hierarchical clustering heatmap (C), showing SASP gene expression profiles in The Cancer Genome Atlas (TCGA)-LUAD cohort. (D–E) Gene Ontology (GO) (D) and Kyoto Encyclopedia of Genes and Genomes (KEGG) (E) enrichment analyses highlighting the top 20 pathways associated with differentially expressed SASP genes. (F) Classification of LUAD patients into two subtypes using Non-negative Matrix Factorization (NMF) based on SASP gene expression patterns. (G) Kaplan–Meier survival analysis comparing overall survival (OS) between the two subtypes. (H) Pie chart illustrating the relationship between SASP subtypes and clinicopathological characteristics.

**Figure 2:**
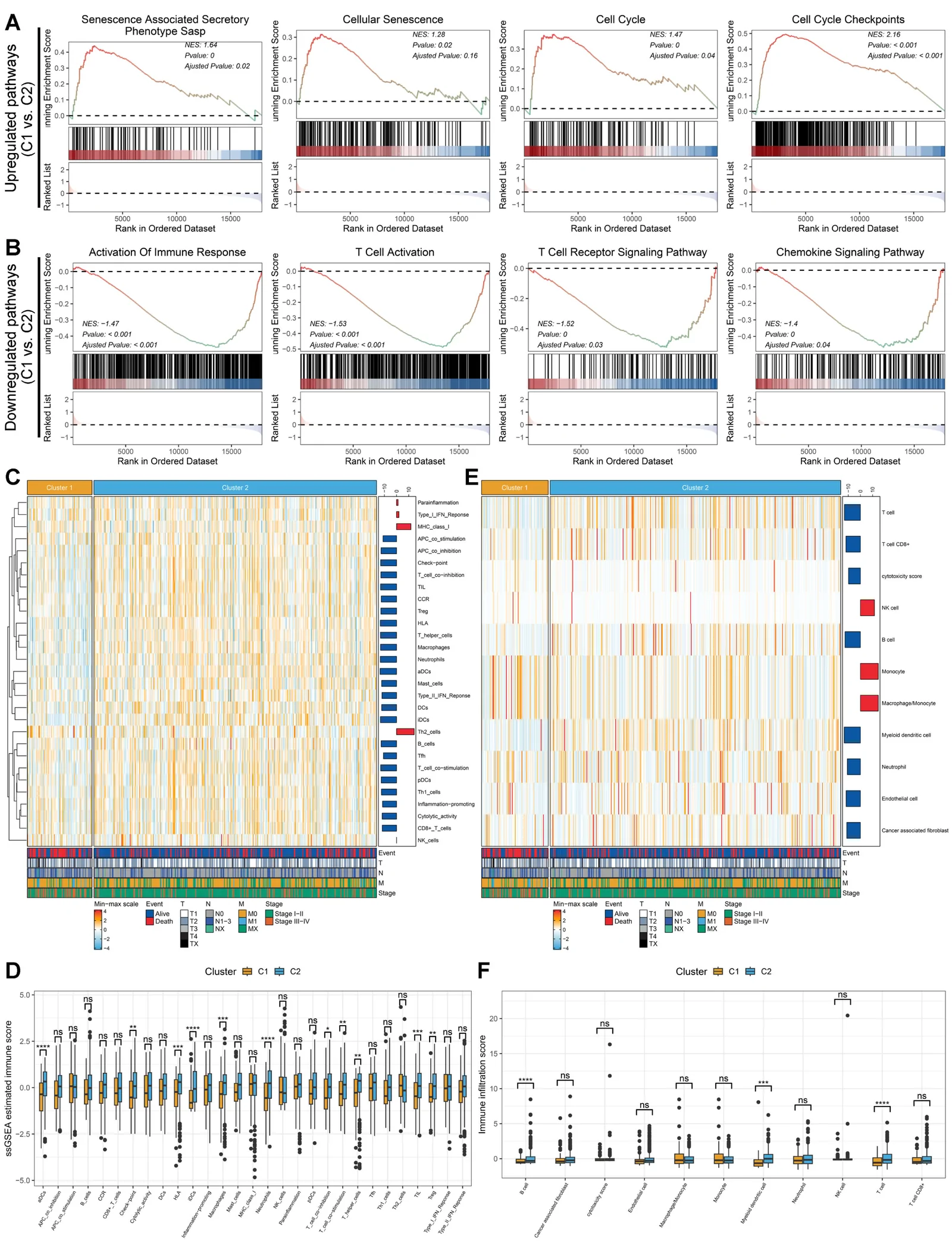
Investigation of biological functions and immune infiltration in distinct senescence-associated secretory phenotype (SASP) subtypes of lung adenocarcinoma (LUAD). (A) Gene Set Enrichment Analysis (GSEA) demonstrates significant activation of biological processes related to SASP, cellular senescence, and the cell cycle in Cluster 1 relative to Cluster 2 among LUAD patients. (B) GSEA reveals significant impairment in processes associated with T cell activation and antitumor immunity in Cluster 1 compared to Cluster 2. (C–D) A clustering heatmap and statistical analysis chart utilizing single-sample GSEA (ssGSEA) estimate immune cell infiltration and immune function across Cluster 1 and Cluster 2 in LUAD patients. (E–F) A clustering heatmap and statistical analysis chart using the MCPcounter algorithm evaluate immune cell infiltration and immune functions within the LUAD subtypes.

### Identification and validation of prognostically relevant SASP genes in LUAD using machine learning approaches

To identify key SASP genes associated with LUAD prognosis, we applied multiple machine learning algorithms. Initially, LASSO regression combined with 10-fold cross-validation was performed to identify prognostically relevant SASP genes (Fig. 3A). This approach identified 16 SASP genes with non-zero coefficients, as shown in Fig. 3B. Subsequently, Random Forest analysis was employed to assess the variable importance of SASP genes, revealing eight key prognostic SASP genes (Fig. 3C). Similarly, XGBoost analysis identified the top 10 SASP genes relevant to prognosis (Fig. 3D). Integrating these results across the three machine learning methods, five core SASP genes were consistently identified, including *ANGPTL4*, *CDKN2A*, *H2AX*, *MMP1*, and *CCL20* (Fig. 3E). The mRNA expression levels of these core genes were then examined in LUAD tumor tissues *versus* adjacent normal tissues. Both unpaired and paired analyses demonstrated significantly higher expression levels of *ANGPTL4*, *CDKN2A*, *H2AX*, *MMP1*, and *CCL20* in LUAD samples compared to adjacent normal tissues (Figs. 3F, 3G). Survival analysis, stratified by the median expression level of these genes, revealed that higher expression of *ANGPTL4*, *H2AX*, *MMP1*, and *CCL20* was associated with poorer OS in LUAD, while *CDKN2A* showed no significant association (Fig. S2). Analysis of the CPTAC proteomic dataset showed higher protein abundance of ANGPTL4, CDKN2A, H2AX, MMP1, and CCL20 in LUAD tissues than in non-tumor tissues (Fig. S3). Protein-level survival analyses showed that higher protein abundance of ANGPTL4, MMP1, and CCL20 was associated with shorter OS (Fig. S3). Together, these transcriptomic and proteomic associations supported further characterization of the five selected genes, although they did not establish their causal roles or therapeutic relevance in LUAD.

**Figure 3:**
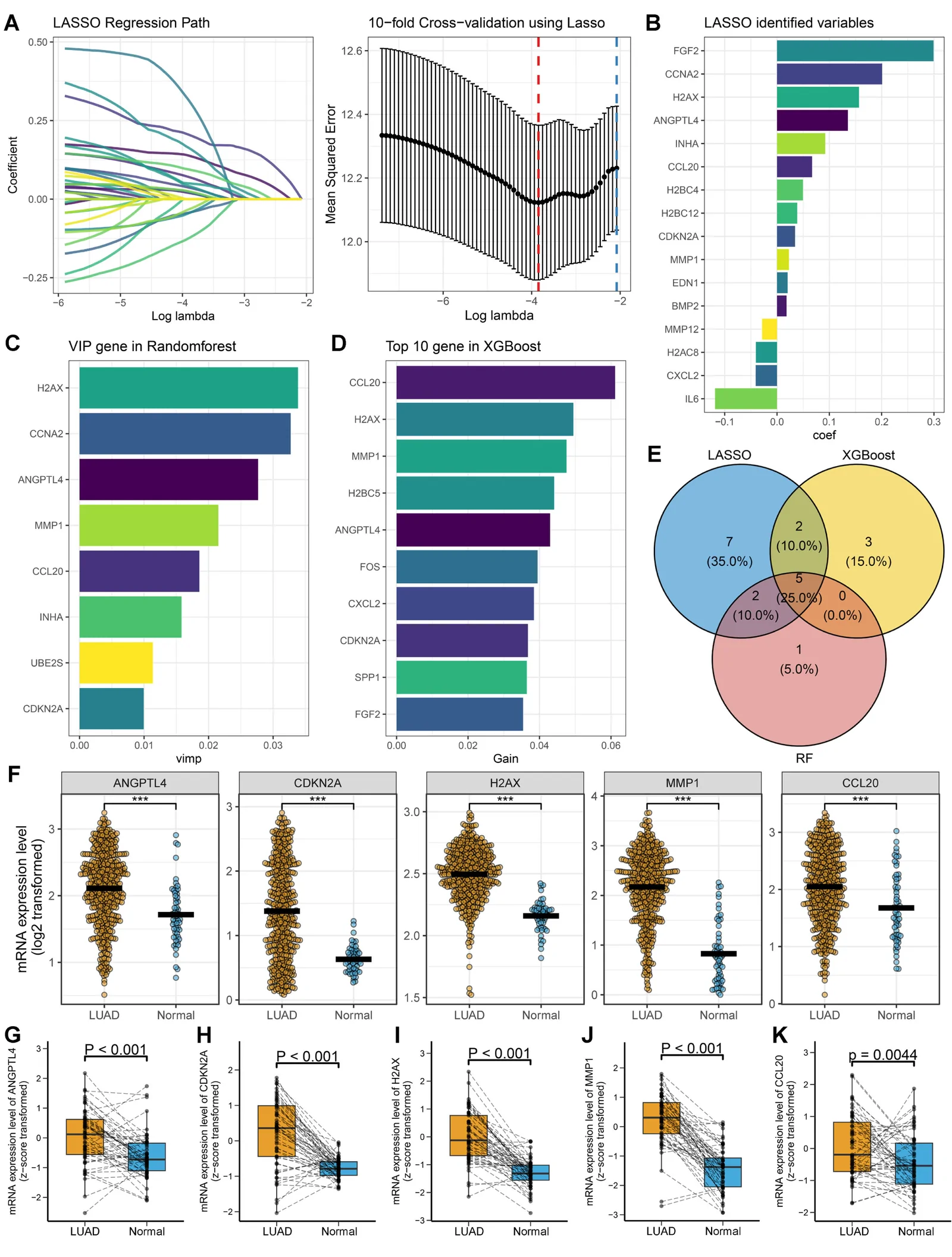
Identification of key senescence-associated secretory phenotype (SASP) genes linked to lung adenocarcinoma (LUAD) prognosis using machine learning algorithms. (A) Least Absolute Shrinkage and Selection Operator (LASSO) regression path and 10-fold cross-validation for identifying key SASP genes. The left panel illustrates the variation in the number of candidate genes with different penalty parameters (Lambda), while the right panel displays the 10-fold cross-validation results. The blue dashed line indicates the number of variables with non-zero regression coefficients at lambda.1se, and the red dashed line indicates the number at lambda.min. (B) Ranking of regression coefficients for variables identified by LASSO. (C) Ranking of key SASP genes based on variable importance from the Random Forest algorithm. (D) Ranking the top 10 SASP genes by importance, identified by the Extreme Gradient Boosting (XGBoost) algorithm. (E) Venn diagram showing the overlap of key SASP genes identified by the three machine learning algorithms, highlighting core SASP genes. (F) Beeswarm plot comparing mRNA expression levels of five key SASP genes in LUAD tumor samples *versus* adjacent normal tissues. (G–K) Box plots with paired analysis comparing the expression levels of the five key SASP genes within LUAD patients.

Subsequently, the expression of selected SASP-related genes was explored in cell lines and clinical specimens. RT-qPCR analysis showed that *ANGPTL4* was markedly upregulated in A549, H1975, and H1299 cells compared with BEAS-2B cells. Because H1299 is generally classified as an NSCLC cell line without definitive LUAD histology, these models are collectively referred to as lung cancer cell lines. *CDKN2A* expression was significantly increased only in H1299 cells, whereas H2AX expression was significantly decreased in A549 and H1975 cells but showed no significant difference in H1299 cells. MMP1 expression exhibited heterogeneous patterns among the LUAD cell lines, with markedly reduced expression in A549 cells, increased expression in H1975 cells, and moderately elevated expression in H1299 cells relative to BEAS-2B cells. Similarly, CCL20 expression was strongly increased in A549 and H1975 cells but was substantially reduced in H1299 cells (Fig. 4A). IHC staining in the examined clinical specimens indicated higher expression of ANGPTL4, CDKN2A, MMP1, and CCL20 in LUAD tumor tissues than in adjacent normal lung tissues, whereas H2AX staining was lower in tumor tissues (Figs. 4B–4F). Thus, the quantitative reverse transcription polymerase chain reaction (RT-qPCR) and IHC findings were not uniformly concordant with the bulk transcriptomic and proteomic results, particularly for *H2AX* and the cell-line expression patterns of *CDKN2A*, *MMP1*, and *CCL20*. These differences may reflect variation in model characteristics, tissue cellular composition, and transcript–protein regulation. Given the limited numbers of cell lines and clinical specimens, these experiments should be considered exploratory expression assessments rather than definitive validation of the five-gene signature. Overall, the five selected SASP-related genes showed prognostic associations and distinct expression patterns across transcriptomic, proteomic, cell-line, and clinical-specimen analyses, providing a basis for subsequent construction and evaluation of the SASP-PRS.

**Figure 4:**
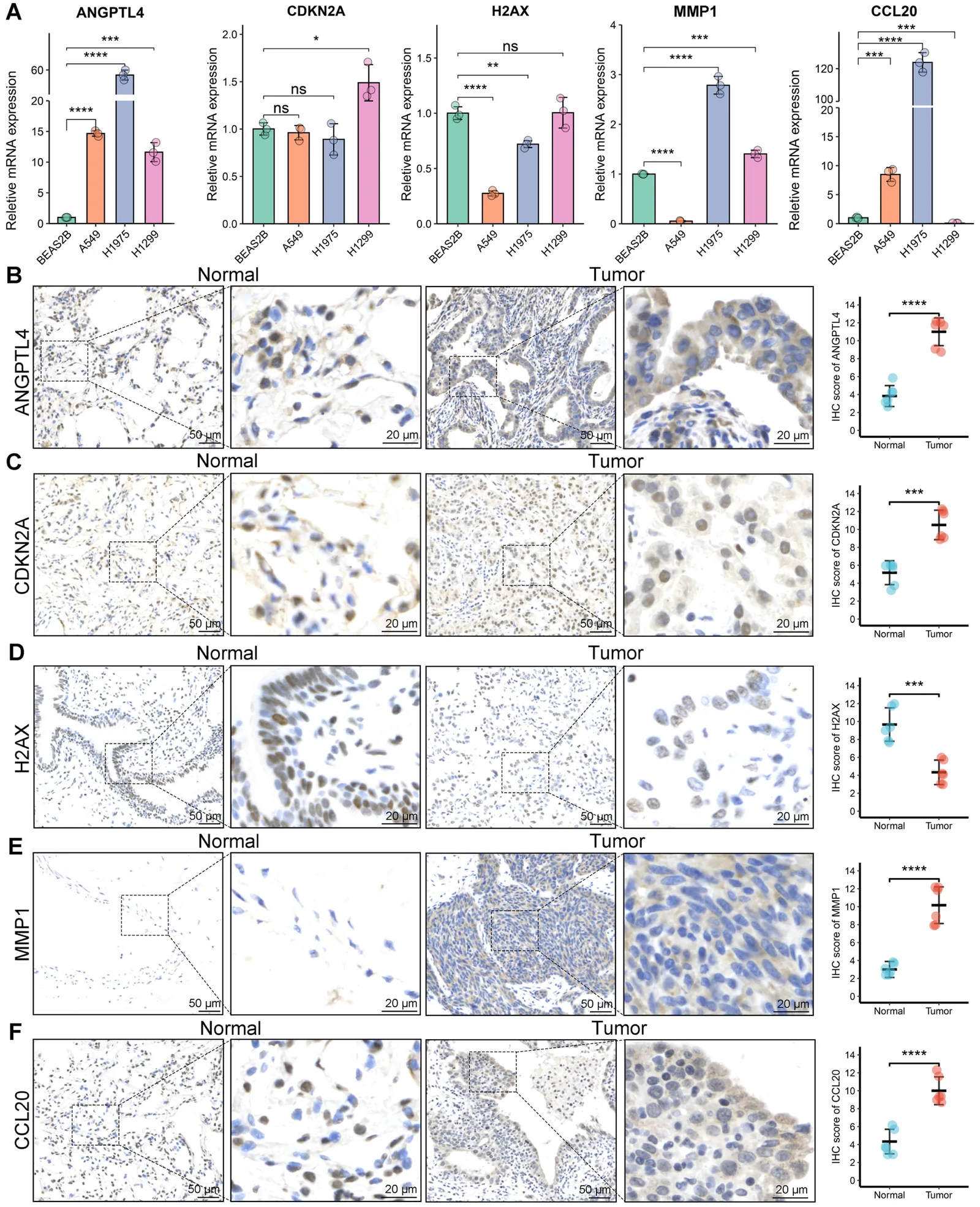
Exploratory expression assessment of senescence-associated secretory phenotype (SASP)-related genes in cell lines and clinical specimens. (A) mRNA expression level of *ANGPTL4*, *CDKN2A*, *H2AX*, *MMP1*, and *CCL20* in different cell lines. (B–F) The protein expression level of ANGPTL4 (B), CDKN2A (C), H2AX (D), MMP1 (E), and CCL20 (F) in lung adenocarcinoma (LUAD) specimens compared to normal samples. *P < 0.05, **P < 0.01, ***P < 0.001, ****P < 0.0001.

### Prognostic value of the SASP-PRS in LUAD

We calculated the SASP-PRS for each patient based on the mRNA expression levels of the identified hub SASP genes, normalizing them into z-scores. Patients in the TCGA-LUAD cohort were stratified into a high SASP-PRS group (standardized SASP-PRS > 0) and a low SASP-PRS group (standardized SASP-PRS ≤ 0). Survival analysis revealed that patients with high SASP-PRS exhibited significantly poorer OS compared to those with low SASP-PRS (Figs. 5A, 5B). Time-dependent ROC analysis demonstrated the acceptable prognostic performance of the SASP-PRS in predicting 1-year (AUC: 0.695), 3-year (AUC: 0.692), and 5-year (AUC: 0.656) OS in the TCGA-LUAD cohort (Fig. 5C). The robustness of the SASP-PRS was validated using the GSE13213 dataset as an independent external LUAD-specific validation cohort. Consistent with the TCGA-LUAD findings, the SASP-PRS exhibited strong discrimination and predictive performance in the GSE13213 cohort, with AUCs of 0.901 for 1-year OS, 0.652 for 3-year OS, and 0.655 for 5-year OS (Figs. 5D–5F). Although the 1-year AUC was relatively high, the moderate AUC values at 3 and 5 years indicate that the predictive performance varied over time and should be interpreted cautiously.

**Figure 5:**
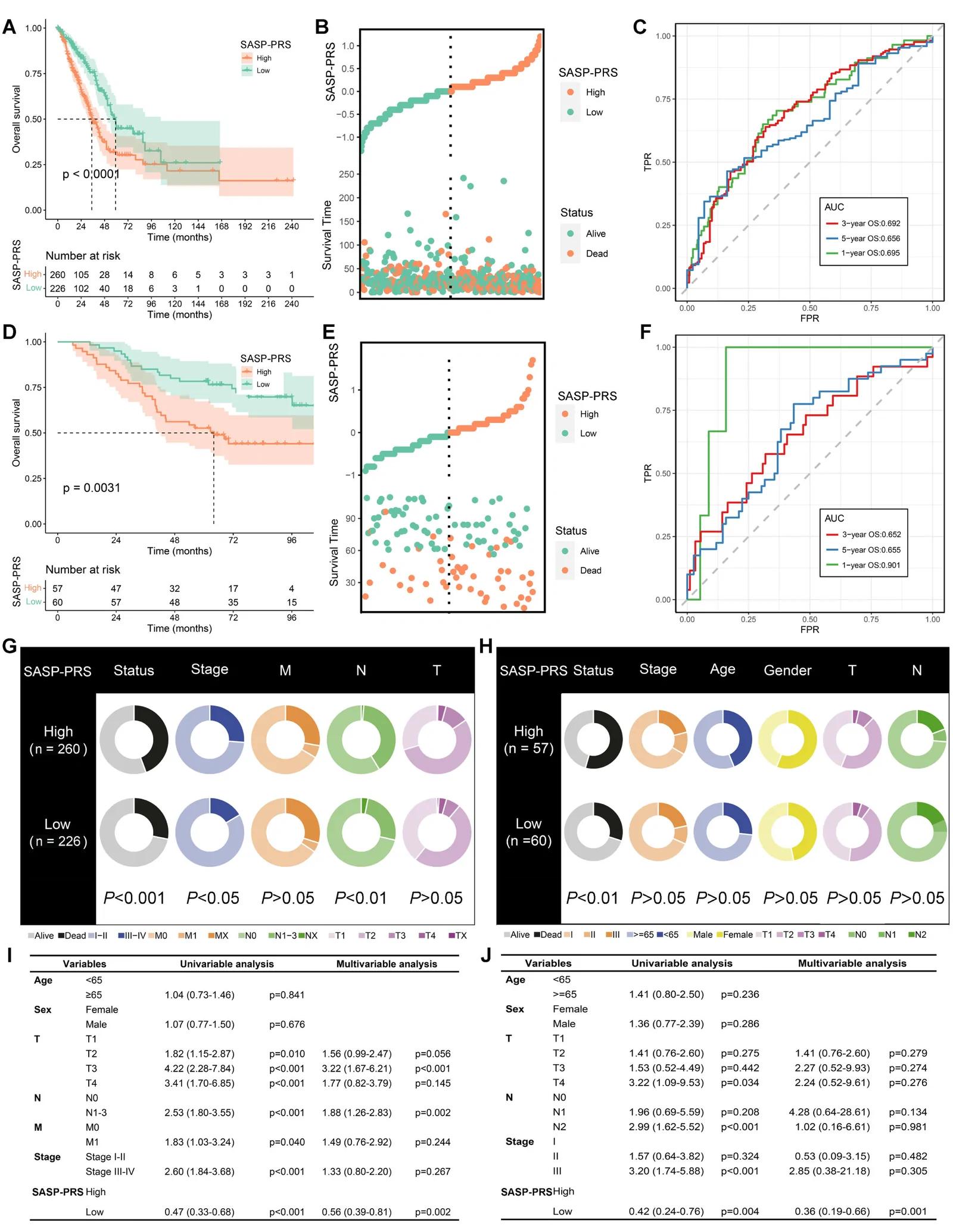
Analysis of the prognostic value of the senescence-associated secretory phenotype (SASP) score in lung adenocarcinoma (LUAD). (A–C) Kaplan–Meier survival curves (A), risk factor plots for the SASP score (B), and time-dependent Receiver Operating Characteristic (ROC) curves predicting 1-, 3-, and 5-year overall survival (OS) (C) in The Cancer Genome Atlas (TCGA)-LUAD cohort. (D–F) Kaplan–Meier survival curves (D), risk factor plots for the SASP score (E), and time-dependent ROC curves predicting 1-, 3-, and 5-year OS in the GSE13213 LUAD cohort (F). (G) Pie charts examining the correlation between the SASP score and clinicopathological parameters in the TCGA-LUAD cohort. (H) Pie charts examining the correlation between the SASP score and clinicopathological parameters in the GSE13213 LUAD cohort. (I) Multivariate Cox regression analysis evaluating the independent prognostic capability of the SASP score in the TCGA-LUAD cohort. (J) Multivariate Cox regression analysis evaluating the independent prognostic capability of the SASP score in the GSE13213 LUAD cohort.

We further examined the relationship between the SASP-PRS and clinicopathological characteristics in the TCGA-LUAD and GSE13213 cohorts. In the TCGA-LUAD cohort, patients with high SASP-PRS were more likely to present with advanced disease stage and N stage (Fig. 5G). However, no significant associations were observed in the GSE13213 dataset (Fig. 5H). Finally, we evaluated the independent prognostic value of the SASP-PRS in the TCGA-LUAD and GSE13213 cohorts. Multivariate Cox regression analysis confirmed the SASP-PRS as an independent prognostic factor for OS in both cohorts (Figs. 5I, 5J). These findings support the prognostic relevance of the SASP-PRS in the analyzed LUAD cohorts; however, additional validation in larger, independent, and preferably prospective LUAD cohorts is required before clinical implementation.

### Cellular characterization of SASP-PRS and its associations with the immune microenvironment and treatment response

We characterized the cellular distribution of SASP-PRS-related activity using scRNA-seq datasets from GSE117570, E-MTAB-6149, and GSE127465. Because these datasets were derived from NSCLC cohorts that were not restricted to LUAD by histological subtype, they were used for supportive cellular-level characterization rather than LUAD-specific validation. After standardized preprocessing (Figs. S4–S6), *t*-SNE analyses demonstrated variation in SASP-PRS-related activity across cell populations (Fig. S7). Malignant or epithelial cells, macrophages, and monocytes showed relatively higher SASP-PRS-related activity than several other cell populations in the analyzed datasets. These observations suggest cellular heterogeneity in the expression of SASP-PRS-related genes but do not establish which cell populations are the primary biological source of the SASP.

We next investigated the complex relationship between the SASP-PRS and various clinical and biological features of LUAD, as summarized in Fig. 6. ssGSEA-based heatmaps showed marked differences in immune infiltration and function between high- and low-SASP groups: the high-SASP group had lower estimated enrichment TILs, DCs, and NK cells and a weaker type II IFN response (Fig. 6A), consistent with ImmuCellAI estimates (Fig. S8). Correlation analyses revealed that the SASP-PRS was negatively associated with immune checkpoint genes *BTLA*, *CD40LG*, *ENTPD1*, *ICOS*, *NCR3*, and *TNFSF14*, and positively with *CD276* and *TNFRSF18* (Fig. 6B). Chemokine analysis showed negative correlations with *CCL13*, *CCL19*, and *CXCL12* (Fig. 6C). GSEA further highlighted the biological processes associated with the SASP-PRS. Patients with high SASP-PRS exhibited substantial activation of pathways related to cellular senescence, SASP, and the cell cycle (Fig. 6D). In contrast, antitumor immunity-related pathways were significantly suppressed in these patients compared to those with low SASP-PRS (Fig. 6E).

**Figure 6:**
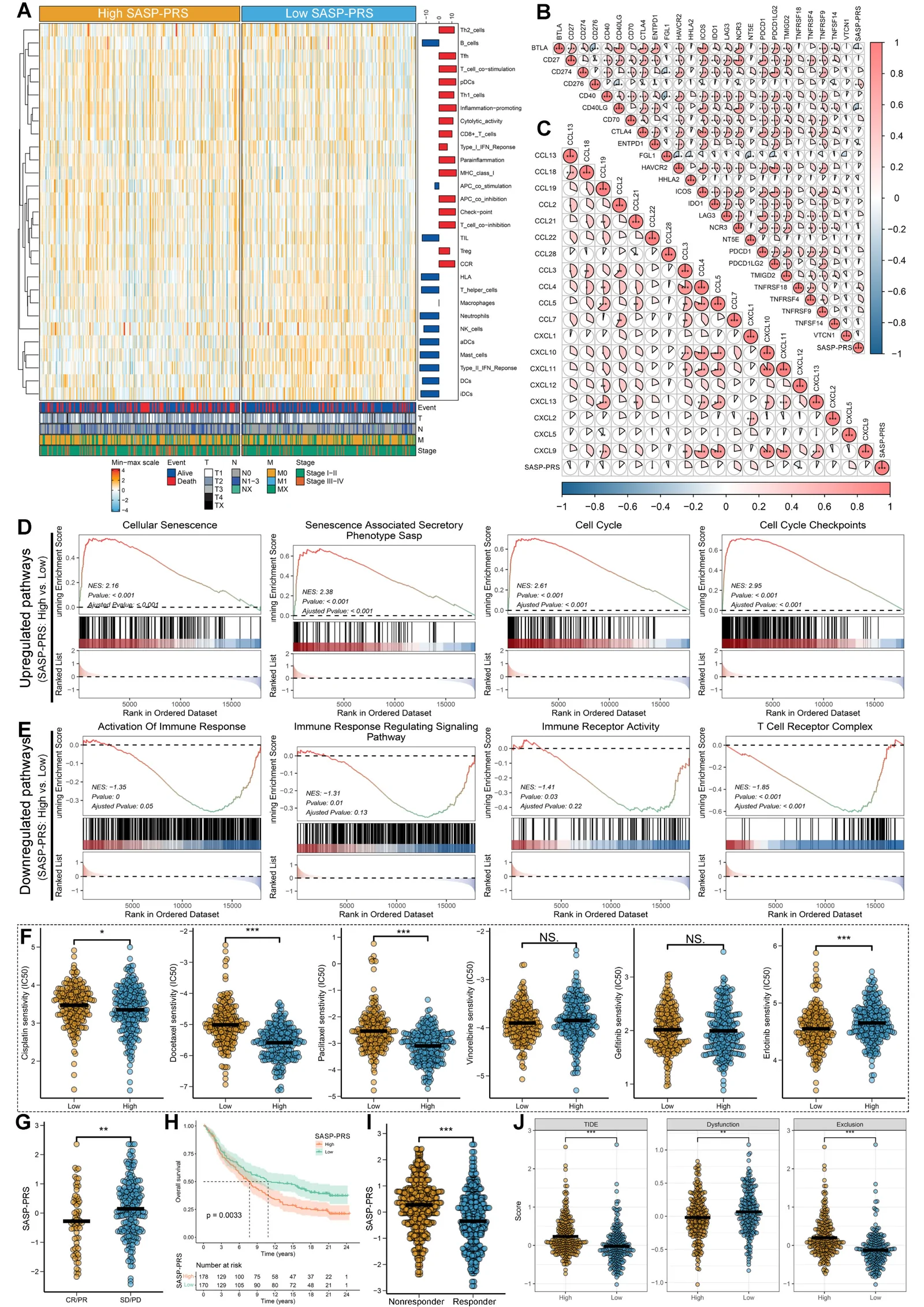
Exploration of the relationship between the senescence-associated secretory phenotype (SASP) score, immune microenvironment, biological functions, and treatment response. (A) Hierarchical clustering heatmap from single-sample Gene Set Enrichment Analysis (ssGSEA) comparing immune infiltration and functional differences between high and low SASP score groups in lung adenocarcinoma (LUAD) patients. (B–C) Correlation heatmap depicting the relationship between the SASP score with immune checkpoint-related genes (B) and chemokine-related genes (C). (D) Gene Set Enrichment Analysis (GSEA) showing significant activation of biological processes related to cellular senescence, SASP, and the cell cycle in patients with high SASP scores. (E) GSEA indicates significant suppression of antitumor immunity-related processes in patients with high *versus* low SASP scores. (F) Analysis of the relationship between SASP score and estimated IC50 of various chemotherapeutic and targeted drugs in LUAD. (G) Association between the SASP score and immunotherapy response in the IMvigor 210 cohort. (H) Kaplan–Meier survival curve demonstrating the risk stratification capability of the SASP score in the IMvigor 210 cohort. (I) Relationship between the SASP score and predicted immunotherapy response in LUAD patients. (J) Association between SASP score and estimated TIDE, immune dysfunction, and immune exclusion scores in LUAD patients. NS, not significant; * *P* < 0.05, **P < 0.01, *** *P* < 0.001.

We also explored the association between the SASP-PRS and algorithmically estimated drug-response measures. Patients in the high SASP-PRS group displayed higher sensitivity to chemotherapy agents such as cisplatin, docetaxel, and paclitaxel, while patients in the low SASP-PRS group showed better responses to the targeted drug erlotinib (Fig. 6F). These results represent computational predictions and should not be interpreted as direct evidence of clinical drug sensitivity or treatment efficacy. In IMvigor210, higher SASP-PRS was associated with poorer immunotherapy response (Fig. 6G) and worse OS post-immunotherapy (Fig. 6H). Additionally, we analyzed the relationship between the SASP-PRS and TIDE scores in the TCGA-LUAD cohort. Higher SASP-PRS was linked to immunotherapy non-responder status (Fig. 6I) and was positively correlated with both TIDE scores and immune exclusion scores (Fig. 6J), suggesting immune escape and impaired immunotherapy efficacy. Furthermore, we analyzed the prognostic value of the SASP-PRS and its relationship with immunotherapy response in a NSCLC immunotherapy cohort. Consistent with our previous findings, a high SASP-PRS score was associated with poorer prognosis among NSCLC patients receiving immunotherapy in the GSE135222 cohort (Fig. S9A). Moreover, the AUC values for predicting progression-free survival (PFS) at 3 months, 6 months, and 12 months post-immunotherapy were 0.865, 0.857, and 0.794, respectively (Fig. S9B). Finally, we found that NSCLC patients with low SASP-PRS scores exhibited a significantly higher rate of durable clinical benefit (DCB) following immunotherapy compared to those with high scores (Fig. S9C). Given the small sample size and the inclusion of NSCLC cases not restricted to LUAD, these findings should be considered exploratory and supportive. They require confirmation in larger cohorts of histologically confirmed LUAD patients receiving immunotherapy.

### Development and evaluation of a nomogram for predicting OS in LUAD patients

We developed a nomogram integrating SASP-PRS with clinical characteristics, including T stage and N stage, to estimate 1-, 3-, and 5-year OS probabilities in patients with LUAD. As illustrated in Fig. 7A, the nomogram combines these prognostic variables to provide individualized survival estimates. Time-dependent ROC curves (Fig. 7B) showed AUC values of 0.753, 0.750, and 0.691 for 1-, 3-, and 5-year OS predictions, respectively. The C-index analysis (Fig. 7C) further indicated the discriminative performance of the model over time. Calibration curves (Fig. 7D) demonstrated an overall agreement between predicted and observed survival probabilities. Kaplan–Meier analysis (Fig. 7E) showed a significant difference in OS between the nomogram-defined high- and low-risk groups (*P* < 0.0001). DCA (Fig. 7F) suggested that the nomogram may provide a net clinical benefit within certain threshold probability ranges. Overall, these findings suggest that the nomogram has potential utility for prognostic assessment in LUAD; however, further validation in independent external cohorts is needed before clinical application.

**Figure 7:**
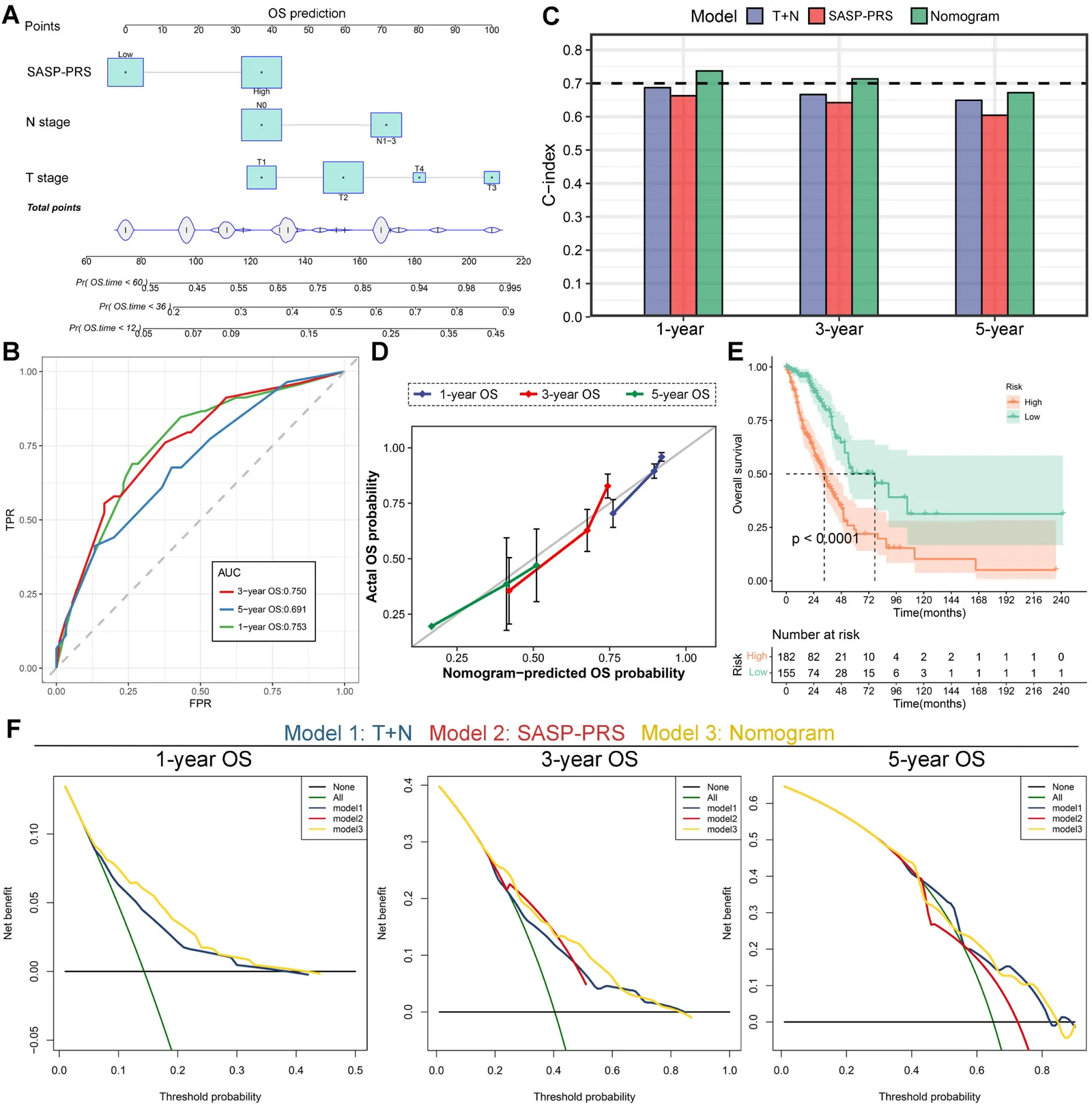
Development and assessment of a nomogram based on the senescence-associated secretory phenotype (SASP) score for predicting the overall survival (OS) of lung adenocarcinoma (LUAD) patients. (A) Nomogram incorporating the SASP score, T stage, and N stage to predict 1-, 3-, and 5-year OS in patients. (B) Time-dependent Receiver Operating Characteristic (ROC) curves demonstrating the discriminatory ability of the nomogram. (C) Time-dependent Concordance Index (C-index) showing the discrimination capability of different models. (D) Calibration curves illustrating the accuracy of the nomogram. (E) Kaplan–Meier survival curve demonstrating the nomogram’s risk stratification capability. (F) Decision curves analysis (DCA) showing the net clinical benefit of different models in predicting OS at various time points in LUAD patients.

## Discussion

This study comprehensively investigated the biological and immunological characteristics of SASP subtypes in LUAD, identifying distinct molecular and immune profiles with potential implications for prognosis and therapeutic strategies. Our findings suggest that SASP-related genes are associated with the TIME, disease progression, and treatment responses in LUAD. We identified two molecular subtypes of LUAD based on SASP gene expression. Cluster 1 was associated with significantly poorer OS and a more immunosuppressive TIME than Cluster 2. Cluster 1 exhibited heightened activation of SASP-related pathways, cellular senescence, and cell cycle processes, along with impaired T cell activation and antitumor immune responses. These findings suggest a potential contribution of SASP-related genes in the phenotypic and functional heterogeneity of LUAD. Immunological profiling revealed that Cluster 1 had reduced estimated infiltration of CD8^+^ T cells, TILs, and DCs, along with a dampened type II IFN response. This immunosuppressive immune contexture may partially contribute to the poorer prognosis associated with Cluster 1, as effective antitumor immune responses are critical for controlling tumor growth and progression. These results align with prior studies demonstrating that SASP factors may modulate the TIME, promote immune evasion, and facilitate tumor progression (Coppé et al., 2010).

Using machine learning approaches, we identified five core SASP genes: *ANGPTL4*, *CDKN2A*, *H2AX*, *MMP1*, and *CCL20*. Exploratory IHC assessment using clinical specimens showed higher protein expression of ANGPTL4, CDKN2A, MMP1, and CCL20 in tumor tissues than in adjacent non-tumor tissues. However, these findings provide protein-level information on individual genes rather than independent validation of the complete SASP-PRS. Notably, some inconsistencies were observed across public datasets, cell-line experiments, and IHC assessment, including the expression pattern of H2AX. The RT-qPCR analysis also revealed heterogeneous expression patterns among the examined LUAD cell lines, which did not uniformly reproduce the patterns observed in clinical transcriptomic and proteomic datasets. These differences may be attributable to the distinct molecular backgrounds and genetic alterations of the cell lines, biological differences between *in vitro* models and clinical tissues, variation in the cellular composition of bulk tumor samples, post-transcriptional regulation, and discrepancies between mRNA- and protein-level measurements. However, the present experiments were not designed to determine whether specific molecular characteristics of the cell lines directly accounted for the observed expression differences. The limited number of cell models and clinical specimens also precludes definitive interpretation of these discrepancies. Accordingly, the RT-qPCR and IHC findings should be regarded as exploratory assessments of individual gene expression rather than definitive experimental validation of the complete SASP-PRS.

*ANGPTL4* is implicated in angiogenesis and metastatic progression and is often observed in advanced-stage tumors, making it a potential therapeutic target for limiting tumor spread (Park et al., 2024). Additionally, *ANGPTL4* may contribute to an immunosuppressive TIME by recruiting regulatory T cells (Tregs) and M2 subtype tumor-associated macrophages (TAMs) while inhibiting CD8^+^ T cells, thereby promoting cancer metastasis and progression (Park et al., 2024; Zhou et al., 2020). *CDKN2A* regulates the cell cycle, with its loss or mutation often leading to uncontrolled proliferation (Adib et al., 2021). Mutated *CDKN2A* has also been associated with immunotherapy resistance in NSCLC and the formation of immune-cold microenvironments (Gutiontov et al., 2021; Deng et al., 2024). *MMP1* is a known driver of tumor progression and metastasis in various cancers, including lung cancer (Merchant et al., 2017). In fibroblasts, senescence-associated MMP1 expression may create an immunosuppressive niche, further contributing to immunotherapy resistance (Gabasa et al., 2021; Gao et al., 2024). *CCL20*, primarily recognized for its role in leukocyte migration, has been linked to tumor proliferation, angiogenesis, immune suppression, and resistance to therapy (Kwantwi et al., 2025). Importantly, *CCL20* and its receptor *CCR6* play a critical role in shaping interactions within the tumor microenvironment (Kwantwi et al., 2025). Within the TIME, *CCL20* recruits immunosuppressive cells, such as Tregs, TAMs, tumor-associated neutrophils, and MDSCs, promoting immune evasion and tumor progression (Kwantwi et al., 2025). Disrupting the expression or function of these SASP genes may modulate tumor–immune interactions and enhance the efficacy of immunotherapy; however, their specific contributions to the SASP-PRS and their causal roles in LUAD immune regulation remain to be established through mechanistic and clinical studies.

Our analysis demonstrated that a high SASP-PRS was computationally associated with features of an immunosuppressive TIME, as evidenced by reduced estimated infiltration of antitumor immune-cell populations, such as CD8^+^ T cells, NK cells, and DCs. Furthermore, the SASP-PRS was positively correlated with inhibitory immune checkpoint molecules such as *CD276* (*B7-H3*) and *TNFRSF18* (*GITR*), and negatively correlated with immune-activating molecules such as *CD40LG*, *ICOS*, and *TNFSF14*. These findings suggest that tumors with a high SASP-PRS may have an immune contexture less favorable for effective antitumor responses, potentially contributing to poor treatment responses and worse clinical outcomes (Gupta et al., 2023). However, these associations were inferred from transcriptomic data using computational algorithms and were not directly confirmed through histological assessment or other immune-cell profiling approaches. They should therefore not be interpreted as direct evidence of altered immune-cell abundance or function. The observed associations provide a rationale for further investigation of SASP-related pathways and immune checkpoints as potential therapeutic targets in LUAD. For example, YL201, an antibody-drug conjugate targeting B7-H3, has shown controllable safety and promising antitumor activity in solid tumors (Ma et al., 2025). Similarly, TNFRSF18 has been identified as highly expressed in Tregs in lung cancer tissues resistant to anti-PD-1 therapy (Wang et al., 2023). Combining local radiotherapy with anti-TNFRSF18 therapy has been shown to enhance PD-L1 blockade-mediated antitumor effects in preclinical models (Song et al., 2024). These findings highlight the potential relevance of targeting SASP-related genes and immune checkpoints such as *CD276* and *TNFRSF18*. Nevertheless, the present study does not establish that targeting these molecules would improve immunotherapy outcomes in LUAD, and this possibility requires dedicated functional and clinical investigation.

This study also demonstrated the prognostic value of the SASP-PRS in stratifying patient outcomes across diverse cohorts. In both the TCGA-LUAD and GSE13213 datasets, the SASP-PRS was associated with OS, with higher scores correlating with worse outcomes. In the IMvigor210 cohort of patients with urothelial carcinoma receiving immunotherapy (Necchi et al., 2017), the SASP-PRS was associated with prognosis, providing supportive evidence in an ICI-treated population from a different tumor type rather than LUAD-specific validation. Importantly, our analysis of the GSE135222 dataset, which includes data from 27 advanced NSCLC patients treated with anti-PD-1/PD-L1 therapies, complements these findings. In this cohort, a high SASP-PRS score was closely associated with poor prognosis and lower rates of durable clinical benefit following immunotherapy. However, owing to the limited sample size and the inclusion of NSCLC rather than exclusively LUAD cases, these findings should be interpreted as supportive external evidence in a broader NSCLC population rather than validation in LUAD patients. Further validation is required in independent, larger cohorts of histologically confirmed LUAD patients receiving immunotherapy. The SASP-PRS may have potential utility for treatment-response assessment in LUAD. Patients with high SASP-PRS showed lower predicted sensitivity to erlotinib and higher predicted sensitivity to chemotherapeutic agents such as cisplatin, docetaxel, and paclitaxel. Moreover, these patients had lower predicted response rates to immunotherapy, consistent with the immunosuppressive immune features computationally associated with SASP-related genes. Using the TIDE algorithm, high SASP-PRS was associated with immune exclusion, which may partly explain the reduced predicted benefit from ICIs. Nevertheless, these treatment-response findings were generated using computational algorithms and do not constitute direct evidence of clinical drug sensitivity or therapeutic efficacy. Whether modulation of SASP-related genes can enhance immunotherapy sensitivity requires further mechanistic and clinical validation (Xiong et al., 2024).

Additionally, existing studies have identified other prognostic signatures in LUAD that provide valuable context for our findings. For instance, the identification and validation of an autophagy-related long non-coding RNA signature has shown potential as a prognostic biomarker, emphasizing the importance of autophagy in tumor biology and patient outcomes (Jiang et al., 2021). Furthermore, a lipid metabolism-related gene signature has revealed distinct prognostic and treatment patterns for patients with early-stage LUAD, highlighting how metabolic pathways can influence tumor behavior and therapeutic responses (Jiang et al., 2022). A recent multi-omics approach has also identified nuclear division fibroblasts as potential prognostic markers and therapeutic targets in LUAD, illustrating the complexity of the tumor microenvironment and the need for integrative analyses (Cao et al., 2026). These models have demonstrated predictive performance for OS in LUAD patients, achieving AUC values ranging from 0.665 to 0.775, which is comparable to the performance of our SASP-based model. Moving forward, multimodal models integrating complementary molecular signatures with clinical and pathological variables may further improve prognostic prediction. However, their value in patients receiving immunotherapy requires specific evaluation in appropriately designed cohorts.

While this study provides insights into the prognostic and therapeutic relevance of SASP-related genes in LUAD, several limitations should be acknowledged. First, the retrospective design may introduce selection bias; thus, prospective studies are needed to validate the clinical utility of the SASP-PRS. Second, key clinical covariates, including smoking history and mutation status, were unavailable in some public datasets, limiting adjustment for potential confounders in prognostic analyses. Third, although SASP-related genes were associated with the TIME, these associations were primarily inferred from transcriptomic data using computational algorithms, and the mechanisms underlying SASP-mediated immune regulation remain unclear. Fourth, the RT-qPCR and IHC analyses were exploratory. The RT-qPCR experiments included a limited number of LUAD cell lines and showed heterogeneous expression patterns that may reflect their distinct molecular backgrounds. However, the biological basis of this heterogeneity was not mechanistically investigated. The IHC assessment was also conducted in a small clinical cohort and did not uniformly reproduce the expression patterns identified through transcriptomic and proteomic analyses. Therefore, these experiments should not be considered definitive validation of the complete SASP-PRS. Direct histological quantification of immune-cell infiltration, such as CD8+ T cells, macrophages, and dendritic cells, was not performed. Larger cohorts and multiplex immunohistochemistry or immunofluorescence studies are needed to validate the immune composition associated with SASP-related signatures. In addition, several external analyses were performed in cohorts not restricted to LUAD by histology, these findings should be regarded as supportive evidence in broader NSCLC populations or other tumor types and require validation in histologically confirmed LUAD cohorts. Finally, although the SASP-PRS showed good short-term predictive performance in GSE13213, its medium- and long-term performance was moderate and should be interpreted cautiously. Predicted therapeutic responses were derived from computational algorithms and do not constitute direct evidence of clinical drug sensitivity. Future studies integrating multi-omics data with comprehensive clinical and pathological information may further improve the performance and clinical applicability of the SASP-PRS.

## Conclusions

In summary, this study identified SASP-related molecular subtypes in LUAD and suggests that SASP-related gene-expression patterns are associated with the TIME, disease progression, prognosis, and treatment-related outcomes. The SASP-PRS may serve as a potential biomarker for risk stratification and prognostic assessment in LUAD; however, its clinical utility requires prospective validation in independent LUAD cohorts. Its associations with immunotherapy outcomes in broader NSCLC and other tumor types should be considered supportive evidence rather than LUAD-specific validation. The findings also provide insights into the relationship among cellular senescence, immune modulation, and LUAD progression. However, prospective studies, independent LUAD-specific validation, and functional experiments are needed to clarify the underlying mechanisms and evaluate the clinical applicability of the SASP-PRS and SASP-targeted strategies.

##  Supplemental Information

10.7717/peerj.21835/supp-1 Supplemental Information 1 Supplemental Figures and Tables

10.7717/peerj.21835/supp-2 Supplemental Information 2 Raw data

10.7717/peerj.21835/supp-3 Supplemental Information 3 Code

10.7717/peerj.21835/supp-4 Supplemental Information 4 MIQE checklist

## Additional Information and Declarations

### Data Availability

The following information was supplied regarding data availability: The raw data is available in the Supplemental Files. The data is available at ArrayExpress: E-MTAB-6149 - Lambrechts, D., Wauters, E., Boeckx, B. et al. Phenotype molding of stromal cells in the lung tumor microenvironment. Nat Med 24, 1277–1289 (2018). https://doi.org/10.1038/s41591-018-0096-5
- https://www.ebi.ac.uk/biostudies/arrayexpress/studies/E-MTAB-6149
The data is available at bio.tools: IMvigor210 - http://research-pub.gene.com/IMvigor210CoreBiologies/
The public data used in this study were obtained from the TCGA Lung Adenocarcinoma (LUAD) project, TCGA accession/project ID: TCGA-LUAD: - https://portal.gdc.cancer.gov/projects/tcga-luad
The data is available at NCBI GEO: GSE13213, GSE117570, GSE127465, and GSE135222.
