# Supplementary material for: Machine learning–derived intracellular protein–based prognostic model reflecting a senescence-associated secretory phenotype-driven immunosuppressive microenvironment in lung adenocarcinoma
Source: PeerJ. 2026 Oct 21;14:e21835. doi: 10.7717/peerj.21835 (PMC13695421; doi:10.7717/peerj.21835)
Supplement: Supplementary file 1 [file peerj-14-21835-s001.docx]

**Machine learning–derived intracellular protein–based prognostic model reflecting a senescence-associated secretory phenotype -driven immunosuppressive microenvironment in lung adenocarcinoma**

Xiaoning Chang^1,*^, Lihong Jiang^2^, Lingxiao Zhang^3^, Tong Ren^4^, Chaoxin Fan^5^, and Pei Li^6,*^

^1^ Department of Oncology, Xi’an Chang’an District Hospital, Xi’an, Shaanxi, China

^2^ Department of Pathology, Xi’an Chang’an District Hospital, Xi’an, Shaanxi, China

^3^ Department of Medical Oncology, The First Affiliated Hospital of Xi’an Jiaotong University, Xi’an, Shaanxi, China

^4^ Department of Nuclear Medicine, Xi’an No. 3 Hospital, the Affiliated Hospital of Northwest University, Xi’an, Shaanxi, China

^5^ Department of Oncology, Xi’an People’s Hospital (Xi’an Fourth Hospital), Xi’an, Shaanxi, China

^6^ Department of Respiratory Medical Oncology, Shandong First Medical University and Shandong Academy of Medical Sciences, Shandong Cancer Hospital and Institute, Jinan, Shandong, China

**Corresponding Authors:**

Xiaoning Chang, Department of Oncology, Xi’an Chang’an District Hospital, No. 251, West Street, Chang'an District, Xi'an, Shaanxi 710100, China; Email address: [18049247751@163.com](mailto:18049247751@163.com)

Pei Li, Department of Respiratory Medical Oncology, Shandong First Medical University and Shandong Academy of Medical Sciences, Shandong Cancer Hospital and Institute, No.440 Jiyan Road, Jinan, Shandong 250117, China; Email address: lzlp09@126.com

**Figure S1.** Determining the optimal clustering configuration using Non-Negative Matrix Factorization (NMF).

**Figure S2.** Kaplan-Meier survival curves of core Senescence-Associated Secretory Phenotype (SASP)-related genes.

**Figure S3.** Protein expression levels and Kaplan-Meier survival curves of key Senescence-Associated Secretory Phenotype (SASP)-related proteins in lung adenocarcinoma (LUAD) and normal tissue samples.

**Figure S4.** Quality control and expression patterns of key Senescence-Associated Secretory Phenotype (SASP) genes at the single-cell level in the GSE117570 dataset.

**Figure S5.** Quality control and expression patterns of key Senescence-Associated Secretory Phenotype (SASP) genes at the single-cell level in the EMTAB6149 dataset.

**Figure S6.** Quality control and expression patterns of key Senescence-Associated Secretory Phenotype (SASP) genes at the single-cell level in the GSE127465 dataset.

**Figure S7**. Characterization of senescence-associated secretory phenotype (SASP) score activity using single-cell transcriptomic data. **(A-C)** Single-cell t-distributed stochastic neighbor embedding (t-SNE) clustering visualizing SASP scores across various cell types in the GSE117570 **(A)**, EMTAB6149 **(B)**, and GSE127465 **(C)** non-small cell lung cancer (NSCLC) cohorts.

**Figure S8.** Immune cell infiltration landscape, as estimated by the ImmuCellAI algorithm, comparing high and low Senescence-Associated Secretory Phenotype (SASP) score groups.

**Figure S9.** Validation of SASP-PRS in NSCLC Immunotherapy Cohort GSE135222 (n=27). **(A)** Kaplan-Meier survival curves demonstrating the difference in prognosis between patients with high and low SASP-PRS scores. **(B)** AUC values for SASP-PRS in predicting progression-free survival (PFS) at 3, 6, and 12 months following immunotherapy, showcasing the predictive accuracy of the SASP-PRS. **(C)** Comparison of durable clinical benefit (DCB) rates in NSCLC patients, indicating that those with low SASP-PRS scores experience significantly higher clinical benefit rates compared to patients with high scores.

| **Table S1. Summary of publicly available datasets used in this study** **Dataset/cohort** \| **Data type** \| **Cancer type** \| **Sample size** \| **Key features** \| **Main application** \| **Source**; TCGA-LUAD \| Bulk RNA-seq and clinical data \| LUAD \| 495 \| Transcriptomic data with clinicopathological and survival information \| Discovery cohort; identification of SASP-related genes; construction and prognostic evaluation of SASP-PRS \| GDC portal; GSE13213 \| Bulk gene-expression microarray (GPL6480) \| LUAD \| 117 \| Gene annotation was conducted based on the GPL6480 platform; expression and survival data available \| External LUAD-specific validation of SASP-PRS prognostic performance \| GEO; IMvigor210 \| Bulk RNA-seq and immunotherapy clinical data \| Metastatic urothelial carcinoma \| 348 patients \| Normalized expression profiles; anti–PD-L1 (atezolizumab) treatment; response and survival data \| Supportive evaluation of the association between SASP-PRS and immunotherapy response in a non-LUAD ICI-treated cohort \| IMvigor210CoreBiologies R package / EGA; GSE135222 \| Bulk RNA-seq \| Advanced NSCLC \| 27 patients \| Patients treated with anti–PD-1/PD-L1 therapies; expression and treatment-response data \| Supportive validation of immunotherapy response prediction in advanced NSCLC (not restricted to LUAD) \| GEO; CPTAC \| Proteomics and clinical annotations \| LUAD \| 106 \| Protein abundance profiles with clinical information \| Protein-level validation of SASP regulators and SASP-PRS \| CPTAC/PDC; GSE117570 \| Single-cell RNA-seq \| NSCLC \| 4 NSCLC 4 Normal \| Single-cell expression profiles of tumor microenvironment \| Supportive cellular-level characterization; not LUAD-specific validation \| GEO; E-MTAB-6149 \| Single-cell RNA-seq \| NSCLC \| 5 \| Single-cell transcriptional profiles across tumor and stromal/immune cells \| Supportive cellular-level characterization; not LUAD-specific validation \| ArrayExpress; GSE127465 \| Single-cell RNA-seq \| NSCLC \| 7 \| Single-cell profiles of tumor and immune cell populations \| Supportive cellular-level characterization; not LUAD-specific validation \| GEO Abbreviations: LUAD, lung adenocarcinoma; NSCLC, non-small cell lung cancer; SASP, senescence-associated secretory phenotype; SASP-PRS, SASP-related prognostic risk score; GDC, Genomic Data Commons; GEO, Gene Expression Omnibus; CPTAC, Clinical Proteomic Tumor Analysis Consortium; PDC, Proteomic Data Commons. **Table S2.** Primer sequences used for quantitative PCR. | |
| --- | --- |
| Primer Name | Sequence (5' to 3') |
| MMP1-FORWARD | TTGGGCTGAAAGTGACTGGGAAAC |
| MMP1-REVERSE | CCACATCAGGCACTCCACATCTG |
| H2AX-FORWARD | CCGGCCTCCAGTTCCCAGTG |
| H2AX-REVERSE | CCGCCAGCTCCAGGATCTCAG |
| ANGPTL4-FORWARD | CCAAGCCTGCCCGAAGAAAGAG |
| ANGPTL4-REVERSE | CGGTTGAAGTCCACTGAGCCATC |
| CDKN2A-FORWARD | CCGTGGACCTGGCTGAGGAG |
| CDKN2A-REVERSE | CGGGGATGTCTGAGGGACCTTC |
| CCL20-FORWARD | GCAAGCAACTTTGACTGCTG |
| CCL20-REVERSE | ATTTGCGCACACAGACAACT |
